# A method to reduce ancestry related germline false positives in tumor only somatic variant calling

**DOI:** 10.1186/s12920-017-0296-8

**Published:** 2017-10-19

**Authors:** Rebecca F. Halperin, John D. Carpten, Zarko Manojlovic, Jessica Aldrich, Jonathan Keats, Sara Byron, Winnie S. Liang, Megan Russell, Daniel Enriquez, Ana Claasen, Irene Cherni, Baffour Awuah, Joseph Oppong, Max S. Wicha, Lisa A. Newman, Evelyn Jaigge, Seungchan Kim, David W. Craig

**Affiliations:** 1 Center for Translational Innovation, Translational Genomics Research Institute, Phoenix, AZ USA; 2 Department of Translational Genomics, University of Southern California, Los Angeles, CA USA; 3 Integrated Cancer Division, Translational Genomics Research Institute, Phoenix, AZ USA; 4 Neurogenomics Division, Translational Genomics Research Institute, Phoenix, AZ USA; 5 Henry Ford Health Systems, Detroit, MI USA; 6 University of Michigan, Ann Arbor, MI USA; 7 Komfo Anokye Teaching Hospital, Kumasi, Ghana

**Keywords:** Somatic mutation, Germline variant, Next generation sequencing, Cancer, Precision medicine, Tumor purity, Copy number alterations

## Abstract

**Background:**

Significant clinical and research applications are driving large scale adoption of individualized tumor sequencing in cancer in order to identify tumors-specific mutations. When a matched germline sample is available, somatic mutations may be identified using comparative callers. However, matched germline samples are frequently not available such as with archival tissues, which makes it difficult to distinguish somatic from germline variants. While population databases may be used to filter out known germline variants, recent studies have shown private germline variants result in an inflated false positive rate in unmatched tumor samples, and the number germline false positives in an individual may be related to ancestry.

**Methods:**

First, we examined the relationship between the germline false positives and ancestry. Then we developed and implemented a tumor only caller (LumosVar) that leverages differences in allelic frequency between somatic and germline variants in impure tumors. We used simulated data to systematically examine how copy number alterations, tumor purity, and sequencing depth should affect the sensitivity of our caller. Finally, we evaluated the caller on real data.

**Results:**

We find the germline false-positive rate is significantly higher for individuals of non-European Ancestry largely due to the limited diversity in public polymorphism databases and due to population-specific characteristics such as admixture or recent expansions. Our Bayesian tumor only caller (LumosVar) is able to greatly reduce false positives from private germline variants, and our sensitivity is similar to predictions based on simulated data.

**Conclusions:**

Taken together, our results suggest that studies of individuals of non-European ancestry would most benefit from our approach. However, high sensitivity requires sufficiently impure tumors and adequate sequencing depth. Even in impure tumors, there are copy number alterations that result in germline and somatic variants having similar allele frequencies, limiting the sensitivity of the approach. We believe our approach could greatly improve the analysis of archival samples in a research setting where the normal is not available.

**Electronic supplementary material:**

The online version of this article (10.1186/s12920-017-0296-8) contains supplementary material, which is available to authorized users.

## Background

Next generation sequencing of tumours is widely used both for discovery of biologically important somatic variants as well as for personalizing treatment based on clinically relevant somatic variants. In both cases, accurate identification of somatic variants is crucial. Ideally, a constitutional DNA sample from the same individual is sequenced in parallel, so that somatic variants can be identified by comparing the tumour to the constitutional sequence. In some cases, the constitutional sample may not be available, such as with many archival samples. Tumour only sequencing is also frequently used in clinical practice [1]. Without a matched germline sequence it is difficult to differentiate between private germline variants and somatic variants [2]. In addition to using variant databases to filter out germline variants, it is also possible to use variant allele frequencies to differentiate between somatic and germline variants [3, 4]. We have implemented a Bayesian tumour-only somatic variant caller, LumosVar, that leverages both prior knowledge of population frequencies of germline and cancer mutations, as well as the observed variant allele frequencies. Here we will evaluate how the tumour content, copy number alterations, read depth, and ancestry of a sample effect the ability to detect somatic variants in an unmatched tumour sample.

Misidentifying germline variants as somatic can be problematic in several ways, depending on the mutation. First, the interpretation of a previously uncharacterized variant would be very different depending on whether it was thought to be somatic or germline. Healthy individuals typically have 130–400 rare non-synonymous germline variants [5]. Therefore, it is not surprising that a study sequencing a panel of 42 cancer genes in 175 participants uncovered 269 germline missense mutations of unknown significance [6]. According to ACMG guidelines, missense germline mutations that are rare and have not been functionally characterized should be reported as uncertain or likely benign [7]. Since tumour suppressors tend to have loss of function mutations through their length, it is unlikely that any specific mutation in a tumour suppressor is well characterized [8]. Clinical tumour sequencing tests, such as MSK-IMPACT, typically include uncharacterized non-synonymous mutations in known cancer genes in the main body of their report [9].

There are some variants, such as those in *BRCA1* and *BRCA2* that are known to occur in the germline and effect cancer risk, but may also occur as somatic mutations in tumours. A study of tumour with matched normal sequencing in over 1000 individuals identified known pathogenic germline variants over 2% of the participants [10]. Jones et al. examined the presence of germline false positives when tumour samples were analysed without a matched normal sample [2]. They used standard somatic variant calling tools designed for matched tumour normal pairs with unmatched tumour normal pairs. After filtering out putative germline variants by removing those found in public databases, they found that an average of 65% of the variants called somatic in the unmatched samples were private germline variants. Additionally, strict filtering removed a small number of mutations that were truly somatic. Most strikingly, they found that ~50% of patients in their cohort would have germline false positives in clinically actionable genes.

The advent of next generation sequencing has accelerated the discovery of cancer driver mutations. However, we have now reached a point where most of the common coding driver mutations have already been discovered [8]. Efforts are now turning to uncover rare and noncoding driver mutations [11–13]. One prominent example of a noncoding driver mutations is the TERT promoter mutation [14]. Statistical methods may be used to prioritize somatic noncoding mutations for functional characterization [15, 16]. Germline variants may also contribute to cancer risk and progression [17]. Different statistical models are required to analyze germline and somatic alterations [18]. When a filtering approach is used, the false positive rate is much higher for noncoding variants, as most large scale sequencing projects have focused on coding regions (Additional file 1: Figure S8). Therefore, we believe our approach would be extremely valuable for discovering novel noncoding variants in unmatched tumor cohorts. As private germline variants are potential false positives in tumour only somatic variant calling, the number of private germline variants that an individual has would have a direct impact on the calling precision. The 1000 genomes project found more novel variants in populations of African ancestry compared to those of European ancestry [5]. We would also expect there to be differences in the number of private germline variants between populations of different ancestry.

In this study, we explicitly examine how the number of private germline variants varies with ancestry and present a strategy to reduce false positives due to private germline variants in tumor-only somatic mutation calling. Our strategy uses a model of variant allele frequencies to improve classification of somatic versus germline variants. We estimate allelic copy number and clonal sample fractions to model the expected allele frequency distributions of somatic and germline variants. We use a Bayesian framework that integrates the allele frequency model with prior probabilities of somatic or germline calculated from 1000 genomes population and cancer mutations counts from COSMIC. In order to evaluate our model, we first use simulations to systematically examine the effects of tumour content, copy number, and read depth on the power to detect somatic variants. Then we use in silico dilutions and down-sampling experiments to examine these effects on real data. Finally, we evaluate the tumour only calling approach on tumour samples of different ancestry.

## Methods

### Nucleic acid isolation, library prep, and sequencing

Biospecimens were previously collected under approval by the Western Institutional Review Board (WIRB #20100721; WIRB #20141201; and WIRB #20031485). “Fresh-frozen” tumor biopsy specimens were used for determination of the tumor’s genomic profile and quality assessed for tumor cellularity, necrosis, crush artifact, etc. Constitutional (or inherited) germline variants were determined by sequencing genomic DNA previously isolated from whole-blood provided at the time of biopsy. RNA and DNA were extracted from tumor biopsy specimens using the Qiagen All Prep kit (Qiagen, Germantown, MD). Table 1 provides information regarding patients and samples.

**Table 1:** Samples and sequencing statistics

Sample name	Cancer type	Self reported ancestry	Reads (Millions)	Mean target coverage	% Covered at 100X
GBM6-HA	Glioblastoma	Hispanic	897	553X	95.7
GBM1-EA	Glioblastoma	Caucasian	2768	2101X	98.3
TNBC3-AA	Breast	African American	905	599X	96.1
TNBC4-AA	Breast	African American	906	546X	95.4
TNBC6-AA	Breast	African American	1040	658X	95.4
TNBC7-AA	Breast	African American	899	588X	95.6
TNBC11-EA	Breast	Caucasian	1066	645X	95.8
TNBC14-EA	Breast	Caucasian	875	454X	94.5
TNBC15-GH	Breast	Ghanian	598	1012X	97.4

Description of the patient samples and sequencing metrics of the patients used in the evaluation dataset. Note that GBM1-EA was sequenced to higher mean target coverage for in-silico dilution and down-sampling experiments. Blood samples from each patient were also sequenced to establish true variant classification

For fresh frozen tissue, tissue from the needle biopsy was disrupted and homogenized in Buffer RLT plus, Qiagen AllPrep DNA/RNA Mini Kit, using the Bullet Blender™, Next Advance. Specifically, tissue was transferred to a microcentrifuge tube containing 600 μl of Buffer RLT plus, and stainless steel beads. The tissue was homogenized in the Bullet Blender at room temperature. The sample was centrifuged at full speed and the lysate was transferred to the Qiagen AllPrep DNA spin column. Genomic DNA purification was conducted as directed by the AllPrep DNA/RNA Mini Handbook, Qiagen. DNA was quantified using the Thermo Scientific Nanodrop spectrophotometer and quality was accessed from 260/280 and 260/230 absorbance ratios.

For blood germline tissue, the QIAamp DNA Blood Maxi Kit, Qiagen, was used to isolate DNA from 8 to 10 ml of whole blood. The protocol was conducted as written. Specifically, the buffy coat layer was isolated from whole blood by centrifugation. The volume of buffy coat was brought up to 5–10 ml with PBS and treated with Qiagen protease at 70 °C. 100% ethanol was added and the sample was applied to a QIAamp Maxi column and centrifuged. Samples were then washed with buffers AW1 and AW2 and eluted in 1000 μl of Buffer AE. The Qubit 2.0 Fluorometer, Invitrogen, and the Nanodrop spectrophotometer, Thermo Scientific, were used to assess DNA quality and concentration.

Slides mounted with 10 um FFPE tissue sections were incubated in a thermal oven overnight at 60 °C. Deparaffinization was conducted on slides by three exchanges of xylene followed by washes in descending concentrations of ethanol (100, 95, 70, 50 and 20%) and a final wash in deionized water. Using a double-edge dissecting needle, tumor tissue was scraped into a 1.5 ml microcentrifuge tube containing 150 μl of Buffer PKD and 10 μl of proteinase K. Samples were vortexed to mix, incubated at 56 °C for 15 min and chilled on ice for 3 min. After centrifugation at 20,000 x g for 15 min, the supernatant was transferred to a new 1.5 ml centrifuge tube for RNA purification. The pellet was resuspended in 180 μl of Buffer ATL containing 40 μl of proteinase K, mixed by vortexing and incubated at 56 °C for 1 h followed by incubation at 90 °C for 2 h. After brief centrifugation, samples were treated with 4 μl of RNAse A (100 mg/ml) at room temperature. Genomic DNA purification was conducted with automation on the QIAcube using the AllPrep DNA/RNA FFPE Kit and the QIAcube standard protocol, Purification of DNA and total RNA including small RNAs from FFPE tissue sections, Version 2 (DNA purification). Samples were eluted in 100 μl of BufferATE. Extracted DNAs were quantified using the Invitrogen 2.0 fluorometer and DNA quality was assessed on the Nanodrop by evaluating 260/280 and 260/230 absorbance ratios.

Captured libraries were sequencing on Illumina HiSeq2500 using paired-end 83x83bp reads. Paired tumor/normal exomes were constructed using KAPA Biosystems’ Library Preparation Kit using the manufacturer’s “with bead” protocol and Agilent’s XT2 adaptors and Agilent’s SureSelect Human All Exon V5 + UTR baits. Unmatched tumor SBS Kit V3 on the Illumina HiSeq.

Tumor and normal genome libraries were sequenced with paired 125 bp reads using Illumina HiSeq2500 V4 chemistry running HCS2.2 controller software and RTA 1.18.61. The five lanes of each library generated 2.819 billion reads for the tumor and 2.968 billion reads for the normal sample.

### Alignment and assembly

Pipeline analysis is triggered when data is written from the sequencer to the analysis server in the form of BCL files. Using a queuing system and write FAIL/COMPLETED system BCL files are converted to FASTQ files (raw sequence) and aligned to the genome using BWA-MEM (version 0.7.8) [19]. BWA-MEM aligns long query sequences against a large reference genome utilizing a backward-search with a Burrows-Wheeler Transform tool. We used the reference genome from 1000 Genomes project build hs37d5 with decoy contigs [b37d5] and Ensembl v74 for annotations [20]. For the samples containing the molecular barcodes, the barcodes were appended to the RG tags using a custom script. BAM files were sorted with SAMTools (version 0.1.2) and merged and duplicate marked using Picard MarkDuplicates.jar (version 1.111). Chastity failed reads were marked in the BAM files through a custom script using the Picard Tools API (version 1.31). Targeted reassembly was performed using ABRA (version 0.94) [21]. These final BAM files were then used to identify genetic variants.

### In silico dilutions and downsampling

For the in silico dilutions, BAMs of the tumor and normal samples were subsampled and then merged using samtools. For the downsampling experiment, samtools was used to subsample the tumor bam.

### Benchmark variant calling

Germline SNV and INDELS were identified using HAPLOTYPE CALLER (version 3.1–1) [22], samtools (version 1.2) [23] and freebayes (v0.9.21) [24] in the constitutional sample. Somatic SNV and INDEL were identified using three different somatic variant callers SEURAT [25], STRELKA [26], and MUTECT [27]. After normalizing INDELs with VT [28], a custom script was used to merge the VCFs from the three callers. A set of ten constitutional samples was pooled as an unmatched reference sample for the somatic SNV and INDEL callers. Agreement of all callers was required to define a true variant (Additional file 2: Table S1). Positions with discordant calls were considered unknown and excluded from sensitivity and precision calculations.

### Germline variant filtering

Known germline variants were identified by their presence in dbSNP (build 146) [29]. Since dbSNP does contain somatic variants, we used the “allele origin” field to exclude those variants from germline filtering. Variants with an allele origin listed as germline or unspecified were considered known germline variants, while those listed as somatic, both, or not present in dbSNP were considered potential somatic or private germline variants. For the filtering approach, variants called by all somatic callers in the pooled reference comparison, and not filtered out as known germline variants were considered somatic calls.

### Simulations

We simulated somatic mutations in order to examine how read depth, tumor content, and copy number, affect the power to detect somatic variants. Read depth of each mutation was drawn from a lognormal distribution where ${\overline{R}}_T$ is the mean target coverage. The standard deviation was derived from fitting a lognormal distribution to the read depth distribution from several tumor samples.

$$ {R}_T\sim lognormal\left({\overline{R}}_T,1,1\right) $$

The expected allele frequency of a somatic variant (ϕ^*S*^) is a function of the fraction of cells in the sample containing the variant (*f*), the total copy number (*N*), and the minor allele copy number (*M*).

1 $$ {\upphi}^S=\frac{f^{\ast}\left(N-M\right)}{f^{\ast }N+{2}^{\ast}\left(1-f\right)} $$

The read depth of the B alleles were drawn from a binomial distribution as *R*_*B*_

$$ {R}_B\sim binomial\left({R}_T,{\upphi}^S\right) $$

### Caller overview

The variant calling approach consists of four main steps. The first step involves analysis of a set of unmatched controls in order to calculate position quality scores and get average exon read depths for the copy number analysis. The second step involves calculating position quality scores of candidate variant positions in the tumor sample, which takes into account both the quality scores from the unmatched controls as well as quality metrics from the tumor sample such as mapping quality scores and strand bias. The third step involves estimating the allelic copy number and clonal sample fractions. The model assumes that due to clonal expansions, subsets of variants will occur in the same fraction of the cells in the sample. There are a fixed number of these subsets (determined by the user – 3 was used in the results presented here) that have the same sample fraction and each variant (mutation or copy number) is assumed to belong to one of these subsets. The user may select any positive integer number of subsets to find. In an unrelated training dataset, we found that three works well for most samples. An expectation maximization approach is used to find the clonal sample fractions that best explain the data and assign the most likely copy number state to each segment and sample fraction to each variant. The fourth step involves finding the posterior probability that each candidate variant is somatic, germline heterozygous, or homozygous based on the expected allelic fractions for somatic and germline variants which takes into account the allelic copy number and clonal sample fractions. The somatic and heterozygous germline variants are then used as input to step three. The caller iterates between step three and step four until the result converges (Additional file 3: Figure S1).

### Position quality scores from unmatched controls

A quality score for each position in the exome is determined based on the assumption that positions that do not appear diploid in control samples are unreliable. The conditional probability of the data (D) given that the position is homozygous (*P*(*D*| *G*_*AA*_)), heterozygous (*P*(*D*| *G*_*AB*_)), or poorly mapped (*P*(*D*| *U*)) are calculated based on the number of reads supporting the B allele (*R*_*B*_), the total number of reads (*R*_*T*_), the mean base quality of the B allele (${Q}_B^b$) and the mean mapping quality of the A or B alleles (${Q}_A^m$or${Q}_B^m$). The read depths of the B allele are assumed to follow a binomial distribution with *R*_*B*_ successes, *R*_*T*_ trials, and the probability of success dependent on the genotype. For homozygous positions, the probability of observing reads supporting the B-allele depends on the mean B allele base quality, and is 0.5 for heterozygous positions.

$$ P\left(D|{G}_{AA}\right)={binomial}_{pmf}\Big({R}_B,{R}_T,{10}^{\frac{-{Q}_B^b}{10}} $$).

*P*(*D*| *G*_*AB*_) = *binomial*_*pmf*_ (*R*_*B*_, *R*_*T*_, 0.5)

$$ P\left(D|U\right)={10}^{\frac{-\mathit{\min}\left({Q}_A^m,{Q}_B^m\right)}{10}} $$

The prior probabilities of homozygous (*π*_*AA*_) or heterozygous (*π*_*AB*_) are based on the population allele frequencies (*F*_*A*_ and *F*_*B*_), assuming Hardy –Weinberg equilibrium and the prior probability that the position is unreliable is a constant (*π*_*U*_) reflecting the percentage of the exome expected to be mappable [30]. The posterior probability that the position is unreliable is given the data:

$$ \mathrm{P}\left(\mathrm{U}|\mathrm{D}\right)=\frac{\mathrm{P}{\left(\mathrm{D}|\mathrm{U}\right)}^{\ast }{\uppi}_U}{P{\left(D|{G}_{AA}\right)}^{\ast }{\uppi}_{AA}+P{\left(D|{G}_{AB}\right)}^{\ast }{\uppi}_{AB}+P{\left(D|U\right)}^{\ast }{\uppi}_U} $$

The mean posterior probability that the position is unreliable is calculated across the unmatched controls for each position and then transformed to Phred-like score.

### Tumor quality metrics and filtering

Sixteen quality metrics are calculated at each position (see Table 2). Each metric has a PASS threshold and a REJECT threshold (see Additional file 4: Table S2). Each position that passes all of the PASS thresholds is assigned to the PASS training group, and each position that meets any of the REJECT criteria is assigned to the REJECT training group. A quadratic discriminant model is fit to the SNVs and Indels separately. All of the positions are classified according to the respective quadratic discriminant model, and the posterior probability of belonging to the PASS group is used to filter the positions on quality.

**Table 2:** Model input parameters

Parameter	Default value or source	Description
K	3	Number of Clones
*f*_*π*_	0.5	Mode of prior distribution of f
*α*_*π*_	1.5	Determines shape of prior distribution of f
*π*(*N* = 0)…*π*(*N* = 3), *π*(*N* ≥ 4)	0.1, 0.15, 0.5, 0.15, 0.1	Copy Number Priors
*π*(*M* = 0), *π*(*M* = 1), *π*(*M* ≥ 2)	[0.25;0.5;0.25]	Minor Allele Copy Number Priors
*α*_*seg*_	1E-5	Segmentation significance cutoff
*ω*	COSMIC	Number of cancer variants observed at the position
*F*_*A*_, *F*_*B*_	1000 Genomes	Population Allele Frequencies
*ρ*_*SNV*_, *ρ*_*indel*_	1E-5, 1E-6	Constant for calculating prior somatic
*F*_*p* − *SNV*_, *F*_*p* − *indel*_	7.14E-5, 1.43E-5	Population allele frequencies assigned to alleles not seen in input population
F_max-somatic_	1E-3	Maximum population allele frequency to be considered a possible somatic variant
${Q}_{min}^m$	10	Minimum mapping quality to count read
${Q}_{min}^b$	5	Minimum base quality to count base
Τ_*PASS*_	0.99	Minimum posterior probability of belonging to the PASS group to be called pass
Τ_*Somatic*_	0.8	Minimum posterior probability of variant is somatic to be called somatic
Τ_*Germline*_	0.8	Minimum posterior probability of variant is germline to be called somatic

Where applicable, default values were determined empirically on an independent data set

### Copy number and clonal sample fraction estimation

In order to properly determine the expected allelic fractions of germline and somatic variants we need to estimate allele specific copy number of clonal and sub-clonal copy number events, as well as the sample fractions of the main clonal and sub-clonal populations. Our model starts with segmented read count data, and finds the most likely allelic copy number state for each segment given the observed mean exon read depths, the B-allele frequencies of the germline heterozygous variants in each segment, and the clonal and sub clonal sample fractions. Using an expectation maximization approach, we find the clonal and sub clonal sample fractions that maximize the probability of the observed mean exon read depths, germline heterozygous B-allele frequencies, as well as somatic variant B-allele frequencies. We also include prior probabilities of copy number states and sample fractions to favor solutions more diploid copy number segments and intermediate sample fractions. The model assumes that at most one clone can have a copy number alteration in a given segment, and the rest of the tumor cells, as well as the normal cells are diploid in that segment. See Table 3 for key notation used in the model.

**Table 3:** Other model parameters and variables

Variable	Descriptions
*Inputs to model*	
R_T_, R_B_	Total read depth, B allele read depth
π_S_, π_AB_, π_AA_	prior probability of somatic, germline heterozygous, germline homozygous variant
${Q}_A^m,{Q}_B^m$	Mean mapping quality of reads supporting the A or B allele
${Q}_B^b$	Mean base quality of bases supporting B allele
X	Total number of exons,
Y	Number of heterozygous germline variants
Z	Number of somatic variants
G	Number of segments
*Parameters fit in maximization*	
f_i_	fraction of cells in the sample with the variants in clone i
C	centering parameter
W	controls the spread of the allelic fraction distributions
*Intermediate variables*	
N	total copy number
M	minor allele copy number
ϕ^S^, ϕ^G^	expected allele fraction of somatic or germline variant
I_S_, I_j_	Index of clonal subset containing somatic variant or copy number variant
Q^*^	Number of copy number altered exons
*Other notation*	
G_AA,_ G_AB_	Germline homozygous or heterozygous genotype
O	Other genotype beside somatic, germline homozygous AA, or germline heterozygous AB
U	Unknown genotype due to poor mapping
i	Index of clonal subset {1, 2, ..., K}
j	Index of segment {1, 2, …, G}
s	Index of somatic variant {1, 2, …, Z}
h	Index of heterozygous variant {1, 2, …, Y}
n	Index of exon {1, 2, …, X}

Key to notation used in describing model

The copy number segmentation is performed on the ratio of the tumor to the normal mean exon read depth using the circular binary segmentation implementation in the Matlab bioinformatics toolbox.

Somatic and germline variant allelic read counts are required to as input to the expectation maximization step. In the initial iteration, likely germline and somatic variants are selected based on database frequencies. In subsequent iterations, the posterior probability described in the “Somatic Variant Calling” section below (Eq. 4) is used to select the positions considered somatic and a similar germline posterior probability is used to select germline variants. In the expectation maximization step, in addition to optimizing the clonal and subclonal sample fractions (*f*), we also optimize a centering parameter (*C*) and a parameter that controls the spread of the allelic fraction distributions (*W*). We aim to maximize the following sum of likelihoods: 1) the likelihood of the exon read depth given the sample fraction and centering parameters, 2) the likelihood of the heterozygous variant B allele read depth given the sample fractions and W parameter, 3) the likelihood of the somatic variant B allele read depth given the sample fractions and *W* parameter. Terms reflecting the prior probabilities of observing the copy number states and sample fractions are also included in the sum to maximize, as shown below. Here *X* is the number of exons, *X* ^*^ is the number of copy number altered exons, *V* is the number of heterozygous germline variants, and *Y* is the number of somatic variants.

$$ \left\{f,W,C\right\}= argmax\left(\frac{\sum_{n=1}^X\mathit{\log}\left(L\left(C,f|{R}_{Tn}\right)\right)+{\sum}_{h=1}^V\mathit{\log}\left(L\left(f,W|{R}_{Bh},{R}_{Th}\right)\right)+{\sum}_{s=1}^Y\mathit{\log}\left(L\left(f,W|{R}_{Bs},{R}_{Ts},\right)\right)+{\sum}_{n=1}^X\mathit{\log}\left(\pi \left({N}_n\right)\right)+{\sum}_{n=1}^X\mathit{\log}\left(\pi \left({M}_n\right)\right)+{\sum}_{s=1}^Y\mathit{\log}\left(\pi \left({f}_s\right)\right)+{\sum}_{n=1}^{X^{\ast }}\mathit{\log}\left(\pi \left({f}_n\right)\right)}{3^{\ast }X+{X}^{\ast }+V+{2}^{\ast }Y}\right) $$

The likelihood of the exon read depth is modeled as a Poisson distribution with a mean of ${\widehat{R}}_{ni}$, which is calculated, based on the observed exon read depths in the unmatched control samples (Eq. 2, below).

$$ L\left(C,{f}_i|{R}_{Tn}\right)={poisson}_{pdf}\left( round\left({R}_{Tn}\right), round\Big({\widehat{R}}_{ni\Big)}\right)\Big) $$

The likelihood of the heterozygous position minor allele read counts (*R*_*Bh*_) are modeled as a beta binomial distribution with an expected allelic fraction ϕ^*G*^ (Eq. 3).

$$ L\left({f}_i,{W}_i|{R}_{Bh},{R}_{Th}\right)={betabinomial}_{pmf}\left({R}_{Bh},{R}_{Th},{W_i}^{\ast }{\upphi}_i,{W}_I\left(1-{\phi}_i\right)\right) $$

The likelihood of somatic position minor allele read counts (*R*_*Bs*_) are modeled as a beta binomial distribution with an expected allelic fraction ϕ^*S*^ (Eq. 1).

$$ L\left(f,W|{R}_{Ts}\right)={betabinomial}_{pmf}\left({R}_{Bs},{R}_{Ts},\mathit{\min}\left({W}_{I_s}{\phi}_{I_s}^S,{W}_i\Big(1-{\phi}_{I_s}^S\right)\right),\mathit{\max}\left({W}_{I_s}{\phi}_{I_s}^S,{W}_{I_s}\Big(1-{\phi}_{I_s}^S\right)\Big) $$

The prior distribution of *f* is described as a beta distribution parameterized such that *f*_*π*_ is the mode of the distribution.

$$ \pi (f)={beta}_{pdf}\left(f,{\alpha}_{\pi },\frac{\alpha_{\pi }-1}{f_{\pi }}-{\alpha}_{\pi }+2\right) $$

In the expectation step, we first estimate the copy number and minor allele copy number of each segment. For each clone (*i*) and segment (*j*), the copy number (*N*_*ij*_) and minor allele copy number (*M*_*ij*_) are calculated based on the mean segment read depth in the tumor ($\overline{R_{Tj}}$) and controls ($\overline{R_{Cj}}$), and the mean segment B allele read depth in likely germline heterozygous positions in the tumor ($\overline{R_{HBj}}$).

$$ {N}_{ij}=\mathit{\max}\left[ round\left(\frac{C^{\ast}\frac{\overline{R_{Tj}}}{\overline{R_{Cj}}}-{2}^{\ast}\left(1-{f}_i\right)}{f_i}\right),0\right] $$

$$ {M}_{ij}= round\left(\frac{{N_{ij}}^{\ast}\overline{R_{HBj}}}{{f_i}^{\ast}\overline{R_{Tj}}}-\frac{1-{f}_i}{2}\right) $$

Then the expected read depth is calculated for each exon (*n*) and clone.

2 $$ {\widehat{R}}_{ni}=\frac{{f_i}^{\ast }{R_{Cn}}^{\ast }{N}_j+{2}^{\ast }{\left(1-{f}_i\right)}^{\ast }{R}_{Cn}}{C} $$

The expected allele frequencies for germline heterozygous positions are also determine for each clone.

3 $$ {\upphi}_{ij}^G=\frac{{f_i}^{\ast }{M}_{ij}}{N_{ij}}+\frac{1-{f}_i}{2} $$

We find the clone I that is most likely to have the copy number alteration in each segment, and then use the expected read depth and expected allele frequency corresponding to the most likely clone for each segment.

$$ {I}_j={argmax}_i\left(\frac{1}{Q_j}\sum_{n=1}^{Q_j}L\left(C,{f}_i|{R}_{Tn}\right)+\frac{1}{V_j}\sum_{h=1}^{V_j}L\left({f}_i,{W}_i|{R}_{Bh},{R}_{Th}\right)\right) $$

We are then able to find expected allele frequencies for each somatic variant, for each sample fraction. If the variant occurs in a copy-altered segment, then we must take into account whether the variant occurs on the major or minor copy of the chromosomal segment. Here we assume that a variant that occurs in the same sample fraction as the copy number alteration will be on the major allele, while variants in other sample fractions would occur on exactly one chromosomal copy.

$$ {\upphi}_{ij}^S=\left\{\begin{array}{c}i={I}_j,\kern0.5em \frac{{f_j}^{\ast}\left({N}_j-{M}_j\right)}{{f_j}^{\ast }{N}_j+{2}^{\ast}\left(1-{f}_j\right)}\ \\ {}i\ne {I}_j\bigwedge {N}_j>0,\kern0.75em \frac{f_i}{{{f_I}_j}^{\ast }{N}_j}+{2}^{\ast}\left(1-{f}_{I_j}\right)\ \\ {}i\ne {I}_j\bigwedge {N}_j=0,\kern0.5em \frac{\mathit{\min}\left(1-{f}_{I_j},\kern0.75em {f}_i\right)\ }{2}\end{array}\right. $$

We can then find the most likely clone for each somatic variant.

$$ {I}_s={argmax}_i\left({betabinomial}_{pmf}\left({R}_{Bs},{R}_{Ts},\mathit{\min}\left({W}_i{\upphi}_{ij}^S,{W}_i\Big(1-{\upphi}_{ij}^S\right)\right),\mathit{\max}\left({W}_i{\upphi}_{ij}^S,{W}_i\Big(1-{\upphi}_{ij}^S\right)\right)\Big) $$

### Somatic variant calling

The somatic variant calling model assumes that reads at a given position were generated based on one of four mutually exclusive models: somatic mutation (*S*), germline heterozygous (*G*_*AB*_), germline homozygous (*G*_*AA*_), or other (*O*). We can then calculate the probability of the data given each of the models.

$$ P\left(\mathrm{D}|\mathrm{S}\right)={\mathrm{max}}_{\mathrm{i}=1\dots \mathrm{K}}\ \left({betabinomial}_{pmf}\left({\mathrm{R}}_B,{\mathrm{R}}_T,\mathit{\min}\left({W}_i{\upphi}_{ij}^S,{W}_i\Big(1-{\upphi}_{ij}^S\right),\mathit{\max}\left({W}_i{\upphi}_{ij}^S,{W}_i\Big(1-{\upphi}_{ij}^S\right)\right)\right) $$

$$ P\left(\mathrm{D}|{G}_{AB}\right)={betabinomial}_{pmf}\left({R}_B,{R}_T,{W_i}^{\ast }{\upphi}_{ij}^G,{W}_i\left(1-{\upphi}_{ij}^G\right)\right) $$

$$ P\left(\mathrm{D}|{G}_{AA}\right)={betabinomial}_{pmf}\left({R}_A,{R}_T,{W_i}^{\ast}\left(1-{10}^{\frac{-{Q}_B^b}{10}}\right),{W_i}^{\ast }{10}^{\frac{-{Q}_B^b}{10}}\right) $$

$$ P\left(\mathrm{D}|\mathrm{O}\right)={betabinomial}_{pmf}\left({R}_T-{R}_A-{R}_B,{R}_T,{W_i}^{\ast}\left(1-{10}^{\frac{-{Q}_A^b}{10}}\right),{W_i}^{\ast }{10}^{\frac{-{Q}_A^b}{10}}\right) $$

The prior probability of a somatic mutation is based on the count of mutations in that position in cosmic (*ω*) and the prior probabilities of the germline genotypes are based on population allele frequencies (*F*_*A*_, *F*_*B*_).

$$ {\pi}_s={\rho}^{\ast}\left(\omega +1\right) $$

$$ {\pi}_{AB}={\left({2}^{\ast }{F_A}^{\ast }{F}_B\right)}^{\ast}\left(1-{\pi}_s\right) $$

$$ {\pi}_{AA}={{F_A}^2}^{\ast}\left(1-{\pi}_s\right) $$

$$ {\pi}_O=1-{\pi}_s-{\pi}_{AB}-{\pi}_{AA} $$

We can then calculate the posterior probability that the mutation is somatic.

4 $$ \mathrm{P}\left(\mathrm{S}|\mathrm{D}\right)=\frac{\mathrm{P}{\left(\mathrm{D}|\mathrm{S}\right)}^{\ast }{\uppi}_S}{P{\left(D|{G}_{AA}\right)}^{\ast }{\uppi}_{AA}+P{\left(D|{G}_{AB}\right)}^{\ast }{\uppi}_{AB}+P{\left(D|S\right)}^{\ast }{\uppi}_S+P{\left(D|O\right)}^{\ast }{\uppi}_O} $$

## Results

**Fig. 1:**
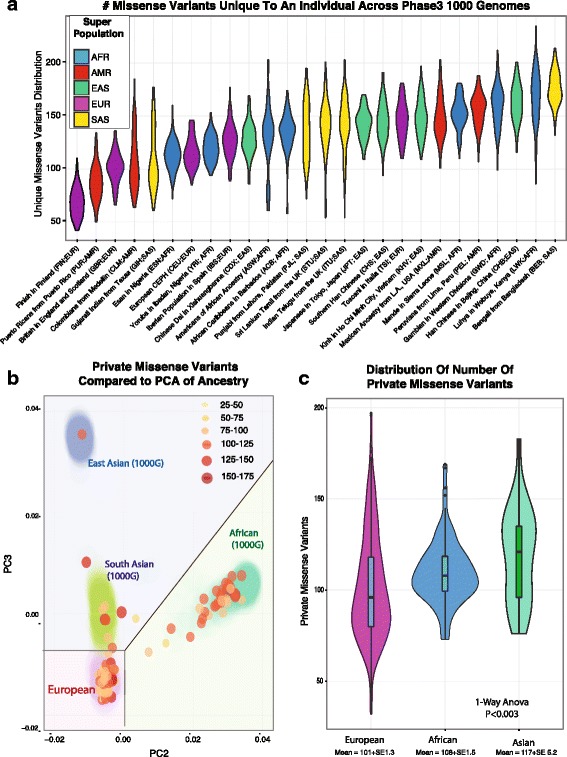
Correlation between ancestry and the effectiveness of using database filters to identify somatic variants. **a** The distribution and number of variants unique to an individual across 2503 individual from Phase 3 of 1000 Genomes plotted as violin plot for each of 26 different populations (indicated by their 3 letter code), and colored based on their ancestral super population. **b** The number of private variants for 150 individuals after filtering through 1000 Genomes, ExAc. not previously sequenced shown by their principle components of common variation (>1%) is shown as a color-metric bubble chart. **c** The distribution of variants within the groups within the right PCA plot, correlating to sections in B, where individuals clustering near those of European, Asian, and African Ancestry

Current approaches for filtering out germline variants from potential somatic variants typically include comparison to databases containing large numbers of germline variants. A recent study has shown increased false positive germline variants in non-Caucasians [3]. We first sought to examine the dependence of private germline variation on ancestry independent of prior databases by utilizing 1000 Genomes Phase 3 data on 26 different populations (Additional file 5: Table S3). In this analysis, for each of the 2503 individuals, germline variants were counted as private if there were found in no other individual within phase 3 of 1000 genomes. Figure 1a shows the distribution of missense variants unique to each individual across the 26 different cohorts. Populations such as Bengali from Bangladesh (BEB) show a significant number of private and rare variants due to both the uniqueness of this population within 1000 Genomes and a rapid recent population expansion. In particularly for the BEB population, there is considerable evidence that one’s ability to precisely distinguish germline and somatic variation would require significantly greater numbers of sequenced individuals than the Finnish population. Evident from the violin plots, admixed populations show a bimodal distribution such as in Americans of African Ancestry in SW (ASW) indicating a high degree of variability. As expected, some populations show a smaller number of unique variants consistent with their geographic isolation such the Puerto Rico participants (PUR).

We extended this analysis by utilizing an additional set of 578 exome sequenced tumor/normal sets not previously included in existing databases. To obtain high quality variant calls, we utilized strict thresholds (increasing marginally false negatives), by excluding genes in highly homologous and paralogous regions, requiring greater than 20X coverage and that they were called by two different germline variant callers (GATK Haplotype Caller and FreeBayes). In this analysis, we limited to single nucleotide variants that have a defined impact on protein transcription or translation, and not found in Phase 3 of 1000 Genomes Phase 3, ExAc 3.0, ESP6500, or ARIC 5600 cohorts. Overall, we find approximately 100 to 200 private variants per individual. We then overlaid ancestry by PCA on common coding variants ascertained from exome sequencing of germline. These are summarized in Fig. 1b and c where one observes there are significantly greater challenges in removing germline false-positives for many populations of non-European ancestry. First, shown in Fig. 1b, the number of private putatively functional variants for each individual are plotted in a bubble graph for the 2nd and 3rd principal components to distinguish non-African samples. The number of private variants for each individual is shown both by color and by size on the bubble chart, and the locations of individuals from 1000 Genomes are shown for orientation. Importantly, this resolution of ancestry shows that even within a European ancestry cohort, there are many individuals who still will have a high number of private variants that would result in a higher number of false positives when detecting somatic variants in tumor only samples by filtering. This effect is seen as we further examine 3 areas of Fig. 1b by grouping individuals heuristically, sectioning off those that cluster near European 1000 Genomes (EUR) for one cluster, those near African 1000 Genomes (AFR) individuals for a second cluster, and those near Eastern and Southern Asian individuals in 1000 Genomes (SAS/EAS). Examining the mean number of missense variants separating into three approximate groups, we see individuals clustering with those of European ancestry with a mean of 101 private missense variants, individuals clustering or admixed with individuals of African ancestry with a mean of 108 missense variants, and those clustering with individuals of South or East Asian ancestry show a mean of 117 missense variants. A 1-way ANOVA analysis between these groups shows significant differences in the number of private variants (*p* < 0.003). These results are overall consistent both within 1000 Genomes populations and within individuals not included with existing databases, showing that individuals of non-European ancestry have greater number of private variants per individual. Still, the wide distribution of individuals of Caucasian ancestry indicates that ancestry alone, as driven by common variation, does not explain all of the variation. Likely admixture with individuals from populations that have recently undergone rapid expansions indicate there is considerably heterogeneity within populations. Taken together, these results are consistent with a lack of diversity being a major but the exclusive factor in the higher number of private variants for individuals of non-European descent. Additional population factors are likely also at play such as the when populations have undergone recent expansions leading to a larger number of variants only within the most recent generations.

The observation that most non-hypermutated cancers have approximately the same number of somatic mutations (~100) as private germline variants has significant implications towards using tumor only sequencing in precision medicine. In Fig. 1c, group 1 would have an approximate 50% false discovery rate with filtering alone, whereas the African group would have over a 70% false discovery rate. Our results suggest filtering-based approaches are substantially more effective for European American individuals. Since using databases to filter germline variants from tumour only somatic variant calls does not appear sufficient, we examine integrating variant allele frequency information.

### Framework for considering allele fraction shifts as a function of copy number and clonal heterogeneity

**Fig. 2:**
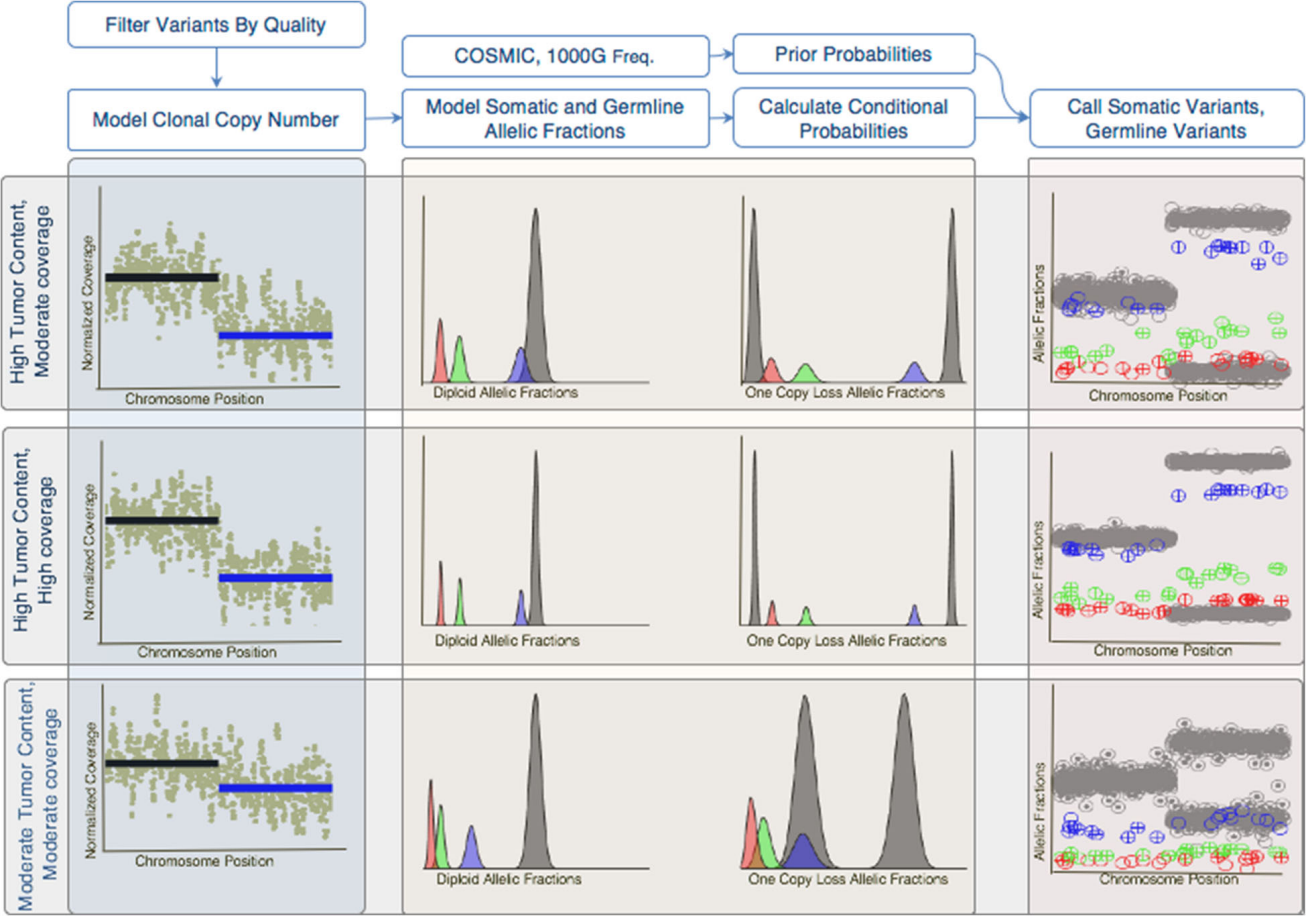
Overview of Variant Calling Strategy. After filtering candidate variant positions by quality, an EM approach is used to fit a model of clonal allelic copy number. The plots on the left show example copy number plots for three conditions, the top panel showing high tumor content and moderate coverage, the middle panels with high tumor content and high coverage, and the bottoms panel with moderate tumor content and moderate coverage. A one copy loss is detected in the segment indicated by the blue line in the first left-most column. Next the expected somatic and germline allelic fractions are modeled in subsequent column. The center two columns plots the expected allelic fractions for germline variants (grey), somatic main clone (blue), and somatic sub clonal (green and red) for diploid regions (left) and one copy loss regions (right). We can see that in high tumor content, moderate coverage, the main clone distribution overlaps with the germline and is difficult to detect in the diploid region, while the red sub-clone is more difficult to detect in the one copy loss region. Increasing the coverage increases sharpness of the distributions making the somatic variants easier to detect. In the moderate tumor content sample, all clones are easy to differentiate from germline in the diploid region, but the main clone is hard to detect in the one copy loss region. Using these distributions to calculate conditional probabilities, as well as using 1000 genomes population frequencies and COSMIC mutation counts to calculate prior probabilities, somatic and germline variants can be called. The right most columns show plots of the allelic fractions of germline (grey) and somatic variants colored by clone. In these, encircled *‘+’* indicates the variant was detected and empty *“o*” indicates a false negative. As expected, in the high tumor content moderate coverage condition, variants in the main clone are detected better in the deleted region, and the number of variants detected increases in the high coverage condition

Since somatic variants will only occur in tumor cells, but germline variants will occur in all cells, we can leverage differences in allele frequencies to differentiate between somatic and normal variants in impure tumor samples. In solid tumours, stromal cells and infiltrating lymphocytes are typically interspersed among the tumour cells [31, 32]. The normal cell contamination in tumours can be leveraged to differentiate somatic from germline variants. For example, in a normal diploid region, a heterozygous germline variant should have an allele frequency around 50% while a heterozygous somatic mutation in an impure tumour should have a lower allele frequency. Still, tumours often have many copy number alterations that will affect the expected allele frequencies of both germline and somatic variants. One approach, implemented by Smith et al., is to fit distribution of allele frequencies of common germline variants in each segment and detect outliers as likely somatic variants [4]. We chose to explicitly model allelic copy number and clonal sample fractions so that we can examine how these factors impact the power to detect somatic variants. A conceptual overview of our approach is shown in Fig. 2 and a more detailed illustration is provided in Additional file 3: Figure S1.

**Fig. 3:**
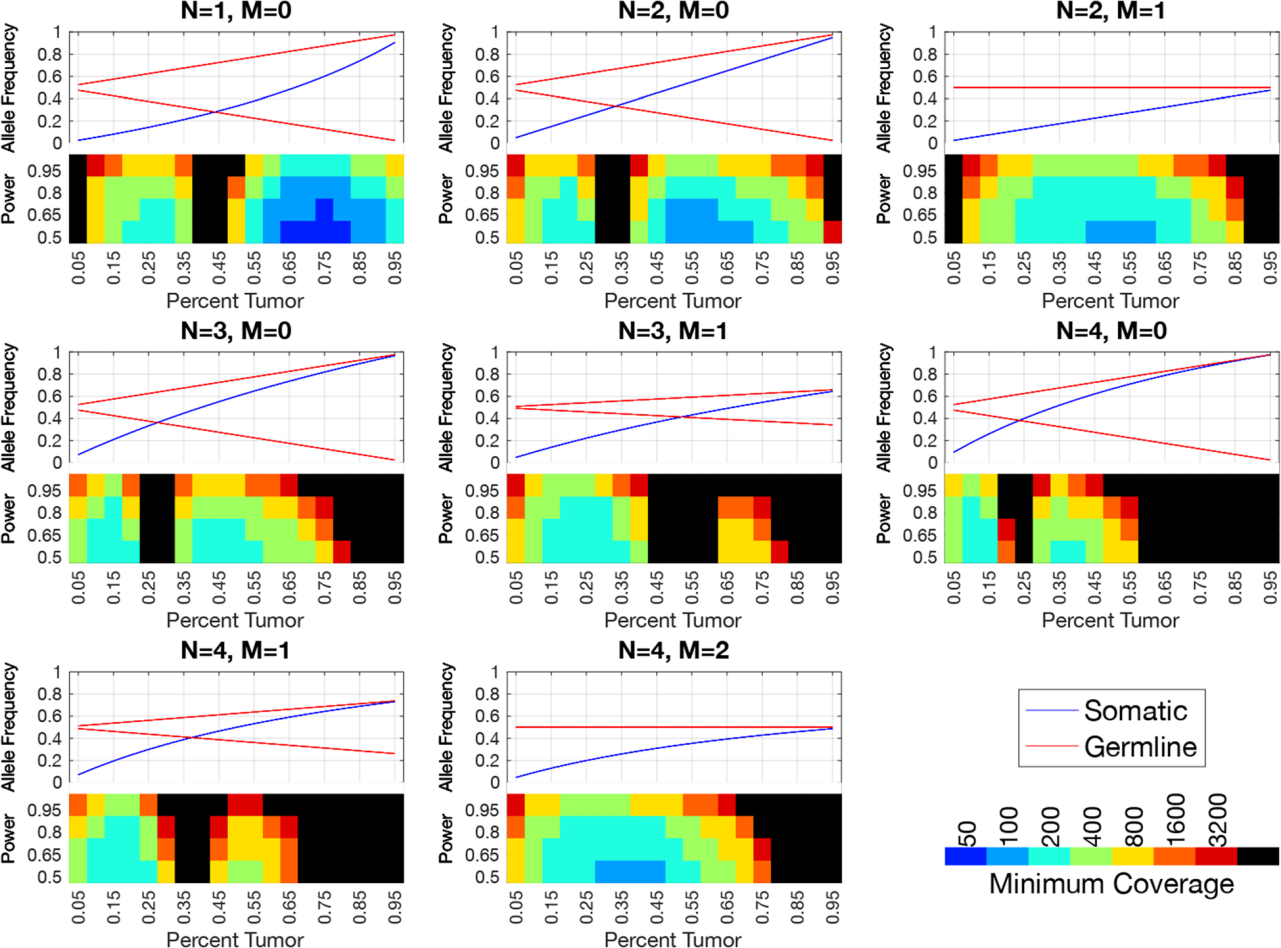
Allele Frequencies of Somatic and Germline Variants and Required Coverage for Somatic Variant Detection by Simulation. The top half of each graph shows the expected allele frequency of somatic (blue) and germline variants (red) by tumor content (x-axis) for different copy number states (plot titles, N indicates total copy number, M indicates minor allele copy number). The bottom half of each graphs shows the coverage required (indicated by the color) to get the power indicated by the y-label. Black squares indicate that the detection power was not achieved even at the highest coverage evaluated. We can see that the closer the somatic and germline allele frequencies, the more difficult it is to detect somatic variants

A key aspect of our strategy is modeling the clonal and subclonal allele specific copy number alterations, which can also effect the allele frequencies of both somatic and germline variants. The expected allele frequencies can be calculated (see methods Eqs. 1 and 3). Figure 3 illustrates how the expected allele frequencies for somatic and germline differ with tumor content for different copy number alterations. As we would expect, the biggest differences in allele frequencies between the somatic and germline variants occurs at the lowest tumor content regardless of copy number state. In a normal diploid region, the difference in allele frequencies monotonically decreases as tumor content increases. However, other copy number states result in points of intermediate tumor content where the allele frequencies for somatic and germline variants are similar. Therefore, we would expect copy number alterations to make it more difficult to detect somatic variants based on allele frequencies.

### Simulations

We would expect that at higher read depth we would be able to measure allele frequency more precisely, therefore would be better able to detect somatic variants. The read depth required should depend on the tumor content and the copy number state. We used simulations to examine how the power to detect somatic variants depends on tumor content, mean target coverage, and copy number state. We simulated somatic variants in eight different copy number states, with sample fraction from 5%–95% and mean target coverage from 50 to 3200 with 1000 variants for each condition. Then we found the percentage that would be called somatic using the default thresholds. We can see in Fig. 3 that the read depth required depends greatly on the sample fraction and copy number state. In a diploid region (*N* = 2,M = 1), we would only need 200X mean target coverage to detect almost 80% of the somatic variants with a sample fraction of 50%. However, we would need 800X mean target coverage to detect a similar proportion of variants with a sample fraction of 75%, and 3200X coverage to detect a similar proportion of variants with a sample fraction of 85%. Copy number alterations reduce the power to detect somatic variants in specific ranges of sample fractions. For example copy neutral loss of heterozygosity (LOH) (N = 2,M = 0) makes it very hard to detect variants with a sample fraction around 35%–40%, while a one copy gain (*N* = 3,M = 1) makes it very hard to detect variants with a sample fraction around 50%–55%.

### Evaluation dataset

A set of nine samples consisting of two glioblastoma samples and seven triple negative breast cancer samples were used to evaluate the tumor only caller. These included four African Americans, three European Americans, one Ghanaian, and one Hispanic. One of the glioblastoma samples was sequenced to 2101X mean target coverage for downsampling and in silico dilution experiments. The other samples were sequenced to 454X-1012X mean target coverage. We used a consensus calling approach to define true somatic and true germline variants, as consensus calling typically outperforms any individual caller [33]. Using strict criteria of detection by three out of three somatic variants callers (or two out of two for indels) we found that each sample had an average of 129 somatic (range 75–196) mutations. We also used three out of three consensus calling to define germline variants, and considered variants private those that did not appear in dbSNP. By these strict criteria, we found an average of 224 (range 126–319) private germline variants per sample.

### Variant quality filtering

Strict quality filtering is required to exclude variants that are not mapped cleanly because any mapping artifacts may shift the measured allele frequency and result in an incorrect classification. Therefore we adopt a two-tiered approach to variant quality filtering. About 80% of somatic variants and 78% of private germline variants have sufficient quality to call (Additional file 6: Figure S2). A much lower percentage of indels meet the strict quality criteria as they are much more difficult to map. We find that increasing coverage increases the number of somatic and private germline variants that pass the strict quality criteria (Additional file 7: Figure S3), while changing the tumor content has little effect (Additional file 8: Figure S4).

### Sample fraction and copy number calling

In the downsampling and in silico dilution experiments, we find that the main copy number events are consistently called, except for at the lowest dilution (Additional file 9: Figure S5). For the one copy loss and LOH events, the sample fraction decreases linearly with the dilution as expected. In our approach, we observe that there is some ambiguity in calling segments as a one copy gain in the highest sample fraction or a higher level gain in a lower sample fraction. Though too small to see in the plot, there is also a ~0.2 megabase deletion on chromosome 9 that is detected as a two copy loss in all but the lowest dilution encompassing CDKN2A. The TNBC samples show a large number of gains and losses (Additional file 10: Figure S6). The large number of copy number alterations are evidence of genome instability which is typical of triple negative breast cancer [34].

### Somatic variant detection sensitivity

**Fig. 4:**
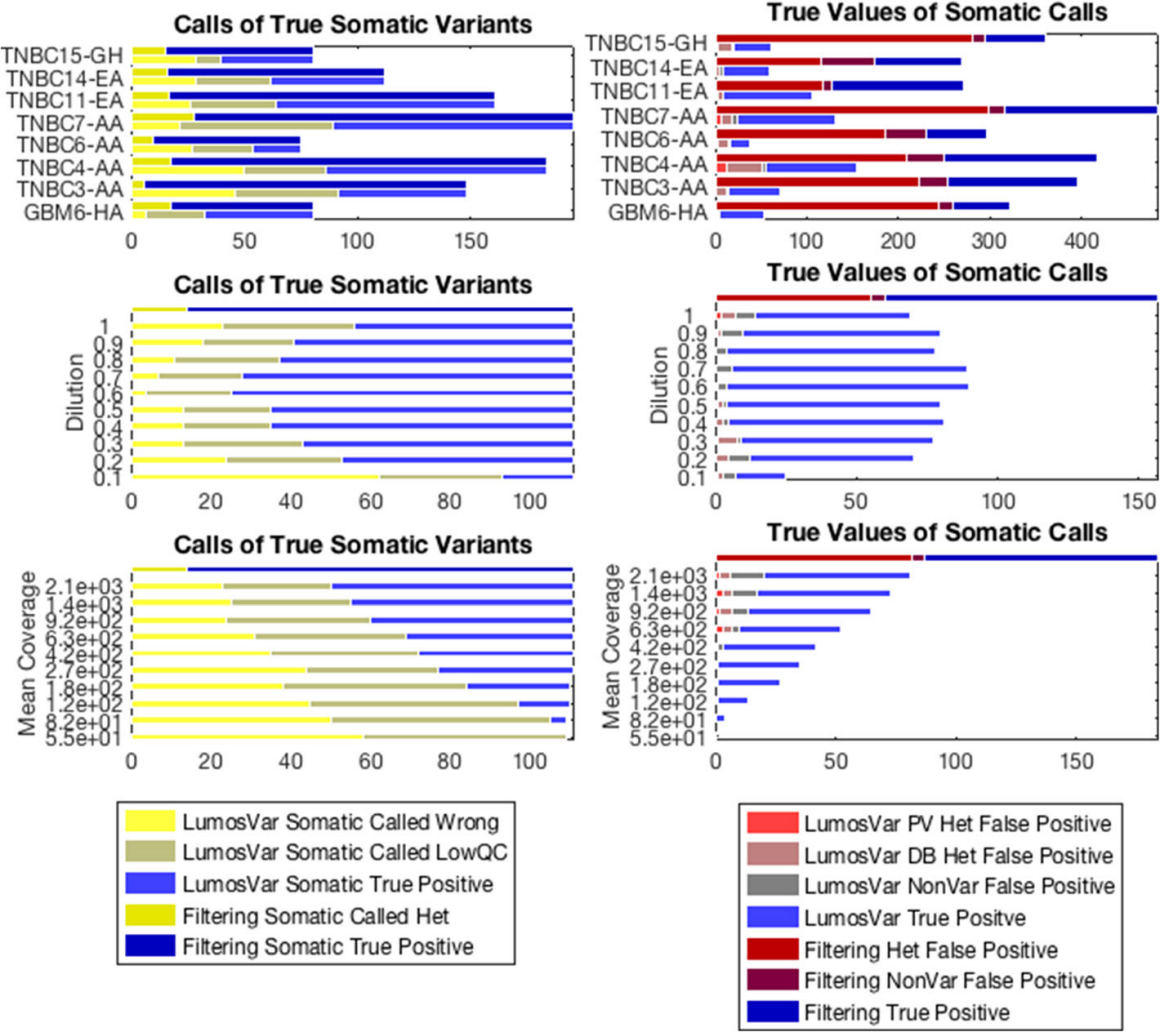
Comparison of Calls of True Somatic Variants and True Values of Variants Called Somatic. The graphs on the left shows the calls of LumosVar (bottom bar in pair) compared to filtering approach (top bar in pair) in calling true somatic variants. The size of the yellow portions of the bars indicate the number of true somatic variants falsely called germline heterozygotes or homozygous, the grey represents true somatic variants that were filtered on quality or not detected as variants, and the blue represents true positive somatic calls. We can see that the filtering approach has better sensitivity (mean TPR 87%, range 78%–96%) compared to the tumor only caller (mean TPR 52%, range 27%–62%). The graphs on the right shows the number of somatic calls by the LumosVar (bottom bar in pair) compared to the filtering approach (top bar in pair) that are truly germline private heterozygous (red), germline heterozygous database variants (pink), homozygous (grey) or truly somatic (blue). We can see that the tumor only caller has better precision (mean PPV 75%, range 56%–89%) compared to the filtering approach (mean PPV 35%, range 19%–55%). The top pair of panels shows the comparison for eight of the nine evaluation samples. The middle of panels shows the comparison for an in-silico dilution series preformed using the ninth evaluation sample (GBMEA1), while the bottom panel shows a down-sampling experiment on the same sample

**Fig. 5:**
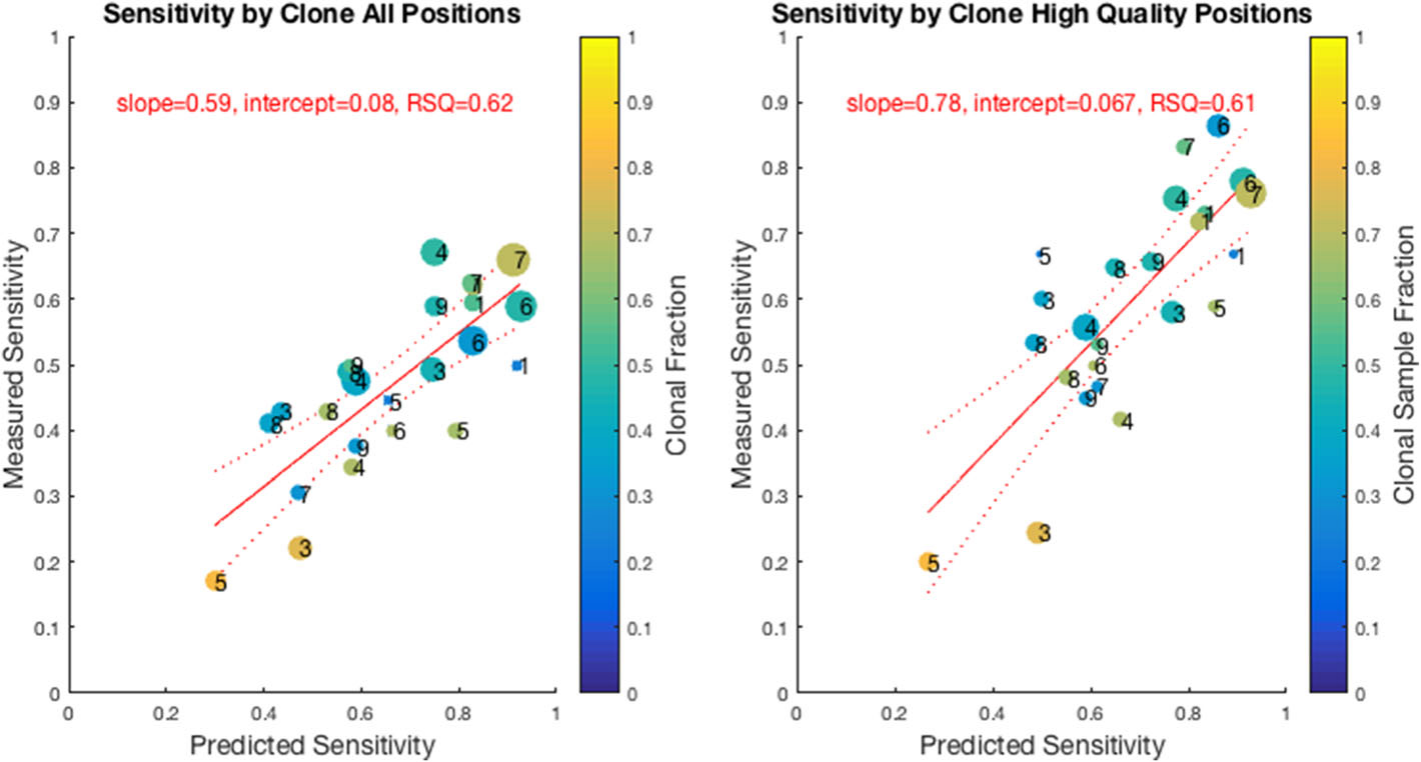
Simulations were used to predict the power to detect each true somatic variant assuming the sample fraction and copy number were correctly called. For each clone and each sample, the true positive rate is plotted against the power predicted from the simulations. The size of the bubble is proportional to the number of true positive variants in each clone, the color the points represents the sample fraction of the clone, and the number indicates the sample number. As expected, the highest sample fraction clone has the worse predicted and observed sensitivity. The graph on the left includes all of the true somatic variants, and the graph on the right only includes those that pass the quality filters. We can see that the predicted power correlates well with the measured sensitivity, particularly when the low quality variants are excluded

Only polymorphic variants appearing in dbSNP are considered false negatives in the filtering approach since the same callers were used to define the truth set, so the sensitivity of the filtering approach does not represent the sensitivity of the callers. There were an average of 16 somatic variants found in dbSNP per sample (range 6–28). LumosVar’s sensitivity varies greatly between samples (Fig. 4). We expect the power to detect somatic variants to depend on the sample fraction, copy number states, and read depth. Using the sample fraction and copy number state assigned to each variant, we simulated somatic variants, and determined proportion of simulated somatic variants that would be called somatic by our model (Fig. 5). These simulations are able to predict very accurately the proportion of somatic variants that we are able to detect, which indicates that the samples with poor sensitivity have copy number states and sample fractions that are not conducive to detecting somatic variants by allele frequency. As we would expect the sensitivity to detect somatic variants increases with coverage (Fig. 4). Also consistent with expectations, we see that the detection sensitivity is best at intermediate tumor content, where the somatic variants would generally have the biggest difference in expected allele frequency from the germline variants (Fig. 4). We also find that we can adjust the caller threshold to tune the tradeoff between sensitivity and precision (Additional file 11: Figure S7).

### Somatic variant detection precision

All of the private germline variants are called as false positives in the filtering approach. Because the number of private germline variants varies by ancestry, the positive predictive value of the filtering approach also depends on ancestry. For the samples of European American ancestry, the positive predictive value of the filtering approach ranges from 35 to 62%, while the samples of Hispanic, African American, or African ancestry the positive predictive value of the filtering approach ranges from 20 to 40%. LumosVar is able to correctly classify most of the private germline variants, and has much better positive predictive (range 67–91%). While there still are some false positive germline variants, many are found in dbSNP (Fig. 3). Combining the filtering approach with the tumor only caller could further improve the positive predictive value.

## Discussion

Private germline variants are difficult to distinguish from somatic variants when a constitutional sample from the same individual is not available. These variants are not present in polymorphism databases, so they may not be easily filtered out. Of critical importance, our results show that the number of private variants is dependent on ancestry. Underlying these differences are under-sampling of some populations within databases along with population-specific characteristics, such as admixture or that have recently undergone rapid expansions.

There are often logistical reasons why only a tissue sample may be available, but the tumor tissue is often a mixture of tumor and surrounding stromal tissue. We demonstrated that a model leveraging deep sequencing to measure differences in allele frequencies between somatic and germline variants can be utilized to call somatic mutations with greater specificity than using population variant frequencies alone. We find that our allele frequency based strategy can reduce by 2/3rds the number of false positives. However, the sensitivity of the allele frequency strategy is highly dependent on the tumor content and the copy number alteration profile of the sample, as well as the sequencing depth. Deep sequencing is important for these models. A minimum sequencing depth of 200-400X is needed with even higher depth required for samples with high tumor content. We believe that the Bayesian calling strategy described here, along with appropriate sample collection and sequencing depth will enable the more accurate detection of somatic variants when the germline samples are not available.

The intuitive question moves to what is the accuracy of tumor only sequencing. It turns out, accuracy is not the most informative statistical tool since one is assured 99% + accuracy due to the millions of true negatives – even if one reports zero variants in a hypermutated sample. Positive predictive value is a natural tool, but it brings forth a different problem. In the case of tumor only sequencing, the positive predictive value for variants called somatic will depend on the number of true mutations. The number of mutations or mutational burden varies by cancer type. Hypermutated phenotypes often seen in melanomas, bladder, and lung cancer can be 100-times higher than the mutational burden scene in lymphomas. Recent data shows the importance of mutational burden as it correlates to the response to immune checkpoint blockade therapies [35]. Given the dependence of mutational burden on cancer type and the relationship between tumor only false positives and ancestry, a more complex picture appears. In some cases, for some ancestries and some cancers can stack in favor of a low false discovery rate. For example, cutaneous melanomas have a higher a mutational burden and are more frequently found in individuals of European ancestry. However, acral melanomas have a low mutational burden and are much more frequent found in individuals of non-European decent (as compared to cutaneous). In this example, a melanoma of a person with non-European decent would show a very low positive predictive value and a European-American would have a higher positive predictive value.

While the goal of the present study was to evaluate the benefit and limitations of leveraging allele frequencies to distinguish somatic and germline variants in unmatched tumor samples, in the process we have developed a tool that we have made available to the research community. We have clearly demonstrated that LumosVar has improved positive predictive value in calling somatic variants compared to database filtering, which is the most commonly used approach with unmatched tumor samples. The sensitivity of LumosVar is clearly too low for us to advocate its use in a clinical setting. While future work could make some improvements in sensitivity (such as through optimizing variant quality filtering), we believe that there are inherent limitations to using allelic fractions to distinguish somatic and germline variants that are clearly demonstrated by our simulations. When high sensitivity and specificity are required in a clinical setting, comparative analysis with a matched germline sample remains the ideal choice. When analyzing archival samples in a research setting, we believe LumosVar would be of great utility.

In addition to calling somatic and germline variants, LumosVar also calls allele specific copy number and assigns both mutations and copy number alterations to clonal sample fractions. There are number of other tools that call allele specific copy number such as exomeCNV [36] and sequenza [37], but these require a tumor/normal pair and do not identify subclones. There are also a number of tools that detect subclonal populations from mutations such as pyClone [38], or from copy number such as Theta [39]. A thorough comparison of lumosVar to these other approaches is beyond the scope of this work, but future work will focus on validating and benchmarking the copy number and clonality functions of LumosVar.

Overall, our results provide insight into how experimental design and sample characteristics can have a large impact on the sensitivity of the allele frequency based tumor only somatic variant detection. Moderate tumor content is optimal and could be achieved through strategic sectioning of FFPE blocks. High sequencing depth is also critical to sensitivity, and as the cost of sequencing continues to decline, high depth sequencing is becoming more common practice. The researcher cannot control the copy number alterations of a tumor, but can be aware that cancer types that stray farther from diploid will be less amenable to this approach. The copy number model assumes that only one type of copy number event may occur in a given segment, which may sometimes be violated, particularly in cancer types with highly unstable genomes. It is possible that the inability of our model to completely capture the complexity of the triple negative breast cancer copy number profiles in our evaluation dataset may have contributed to some of our variant misclassification. However, our impression from visual inspection of misclassified variants due to incorrect copy number calls is due to uncertainty in the placement of segmentation boundaries rather than incorrect assignment of a copy number state within a segment. Since different copy number alterations have tumor content where the somatic and germline variants are most difficult to distinguish, it could be valuable to sequence different sections of the same tumor that may have different tumor content. We intended to extend our model to leverage multiple samples from the same patient.

## Conclusions

The number of germline false positives detected in tumor only sequencing is dependent on the individual’s ancestry. Our Bayesian framework, which integrates modeling copy number and clonality, is able to greatly reduce the number of germline false positives. Sensitivity of our approach depends on tumor purity, coverage and copy number alterations. With appropriate experimental design, our approach has the potential to be extremely useful for somatic variant calling when matched normal tissue is not available, particularly in individuals of non-European ancestry.

### Availability and requirements

LumosVar requires Perl, Samtools, htslib, and MATLAB runtime. The main inputs are bam files, which may be generated by BWA. LumosVar is available for download at https://github.com/tgen/LumosVar.

## Additional files

Additional file 1: Figure S8. Performance by Variant Type. The graphs on the left shows the calls of LumosVar (bottom bar in pair) compared to filtering approach (top bar in pair) in calling true somatic variants. The size of the yellow portions of the bars indicate the number of true somatic variants falsely called germline heterozygotes or homozygous, the grey represents true somatic variants that were filtered on quality or not detected as variants, and the blue represents true positive somatic calls. The graphs on the right shows the number of somatic calls by the LumosVar (bottom bar in pair) compared to the filtering approach (top bar in pair) that are truly germline private heterozygous (red), germline heterozygous database variants (pink), homozygous (grey) or truly somatic (blue). We can see that proportion of false positives in the filtering approach is much higher in non-coding variants than other variant types. (PNG 47 kb)

Additional file 2: Table S1. Definition of True Variants. Describes the criteria for counting a variant as a true variant. Germline variants were called by haplotype caller, samtools, and freebayes. Somatic variants were called by Mutect, Seurat, and Strelka. (DOCX 15 kb)

Additional file 3: Figure S1. Somatic Variant Calling Workflow. Illustrates a detailed workflow of the somatic variant calling process. The steps from “Transpose to pileup” and below are performed by the lumosVar software. (PNG 151 kb)

Additional file 4: Table S2. Filtering Metrics. The criteria used to initially classify a variant in the training set for the quadratic discriminant model. (DOCX 14 kb)

Additional file 5: Table S3. One thousand Genomes Population Codes. Abbreviations used to describe the populations from the 1000 Genomes Project. (DOCX 17 kb)

Additional file 6: Figure S3. Mapping coverage as a function of variant calls. (PNG 38 kb)

Additional file 7: Figure S2. Variant Quality Filtering By Sample. Shows the number of variants of each type, in each quality filtering category. Each graph represents a variant type, each bar represents a sample, and the color of the bar represents the number of variants in each quality category. High quality positions have a PT > 0.99. Low quality positions have a PT < 0.99 but PV > 0.99. Artifacts have a PV < 0.99 and non variants are not considered by the tumor only caller (NaN). (PNG 49 kb)

Additional file 8: Figure S4. Variant Quality Filtering Across Dilutions. Shows the number of variants of each type, in each quality filtering category. Each graph represents a variant type, each bar represents a dilution, and the color of the bar represents the number of variants in each quality category. High quality positions have a PT > 0.99. Low quality positions have a PT < 0.99 but PV > 0.99. Artifacts have a PV < 0.99 and non variants are not considered by the tumor only caller (NaN). (PNG 43 kb)

Additional file 9: Figure S5. Copy Number and Sample Fraction Across Dilutions. The copy number (left), minor allele copy number (center) and sample fraction of the copy number events (right) are plotted as heatmaps. (PNG 35 kb)

Additional file 10: Figure S6. Copy Number of and Sample Fractions Across Sample Set. The copy number (left), minor allele copy number (center) and sample fraction of the copy number events (right) are plotted as heatmaps. (PNG 68 kb)

Additional file 11: Figure S7. Effect of Threshold on Sensitivity and Precision. The true positive rate (left) or positive predictive value (right) is plotted against the pSomatic threshold. Each line represents different mean target coverage (top) or dilution (bottom). Only high trust true somatic or private germline variants are included in this graph. As we would expect, the sensitivity decreases with the threshold, but the positive predictive value increases. We also find that higher coverage results in better sensitivity, but lower positive predictive value. At higher coverage, the threshold may be increased to improve the positive predictive value with less loss sensitivity. (PNG 185 kb)

## Abbreviations

ACMG: American College of Medical Genetics and Genomics
ARIC: Atherosclerosis Risk In Communities
COSMIC: Catalogue of Somatic Mutations in Cancer
dbSNP: The Single Nucleotide Polymorphism database
ESP: NHLBI Exome Sequencing Project
ExAc: Exome Aggregation Consortium
FFPE: Formalin Fixed Paraffin Embedded
INDEL: Insertion or deletion
LOH: Loss of Heterozygosity
SNV: Single Nucleotide Variant
WIRB: Western Institutional Review Board. Also see Additional file 5: Table S3 for 1000 genomes population codes
