# Supplementary figures and images for: A method to reduce ancestry related germline false positives in tumor only somatic variant calling
Source: BMC Med Genomics. 2017 Oct 19;10:61. doi: 10.1186/s12920-017-0296-8 (PMC5649057; doi:10.1186/s12920-017-0296-8)

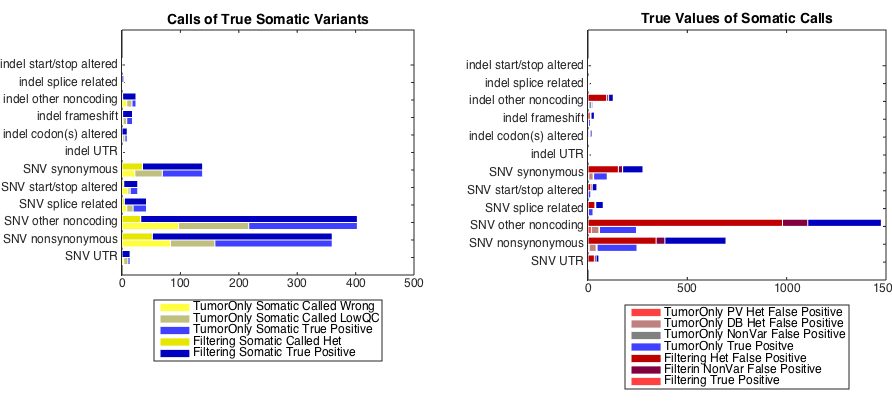

Supplement: Supplementary file 1 — Performance by Variant Type. The graphs on the left shows the calls of LumosVar (bottom bar in pair) compared to filtering approach (top bar in pair) in calling true somatic variants. The size of the yellow portions of the bars indicate the number of true somatic variants falsely called germline heterozygotes or homozygous, the grey represents true somatic variants that were filtered on quality or not detected as variants, and the blue represents true positive somatic calls. The graphs on the right shows the number of somatic calls by the LumosVar (bottom bar in pair) compared to the filtering approach (top bar in pair) that are truly germline private heterozygous (red), germline heterozygous database variants (pink), homozygous (grey) or truly somatic (blue). We can see that proportion of false positives in the filtering approach is much higher in non-coding variants than other variant types. (PNG 47 kb) [file 12920_2017_296_MOESM1_ESM.png]

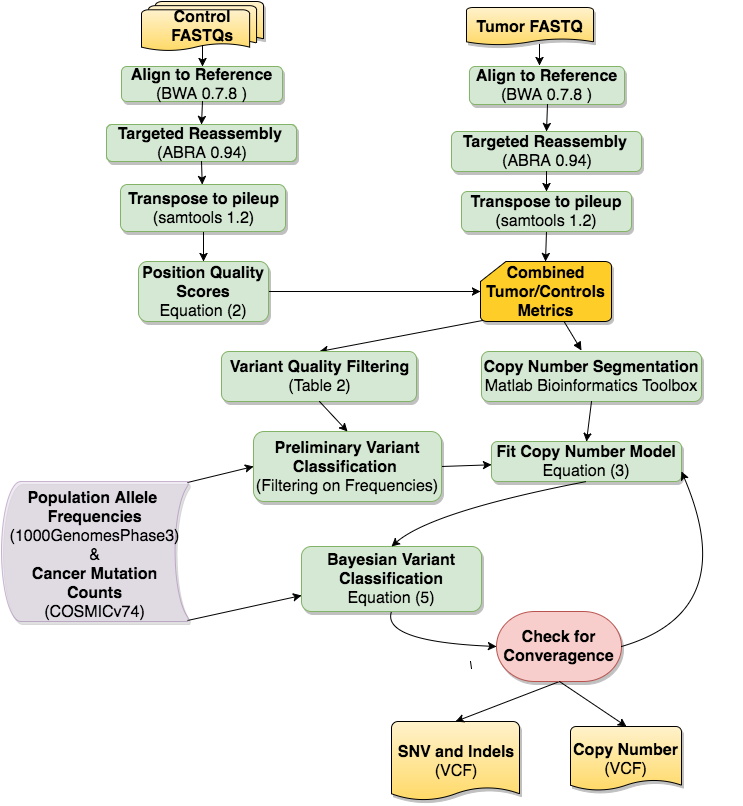

Supplement: Supplementary file 3 — Somatic Variant Calling Workflow. Illustrates a detailed workflow of the somatic variant calling process. The steps from “Transpose to pileup” and below are performed by the lumosVar software. (PNG 151 kb) [file 12920_2017_296_MOESM3_ESM.png]

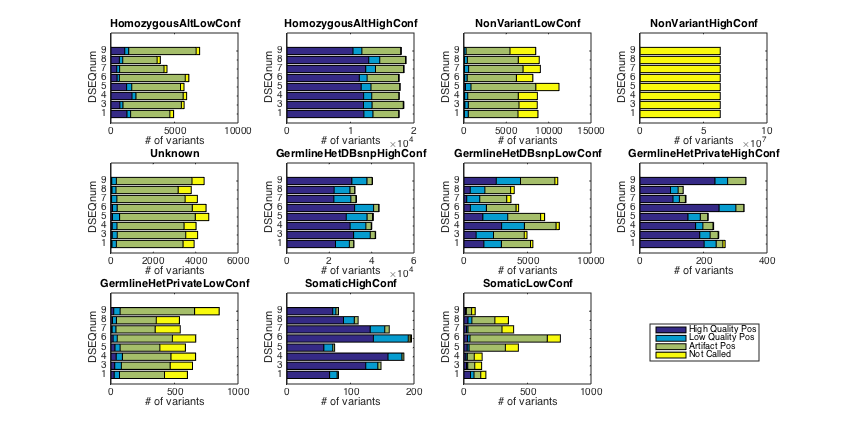

Supplement: Supplementary file 6 — Mapping coverage as a function of variant calls. (PNG 38 kb) [file 12920_2017_296_MOESM6_ESM.png]

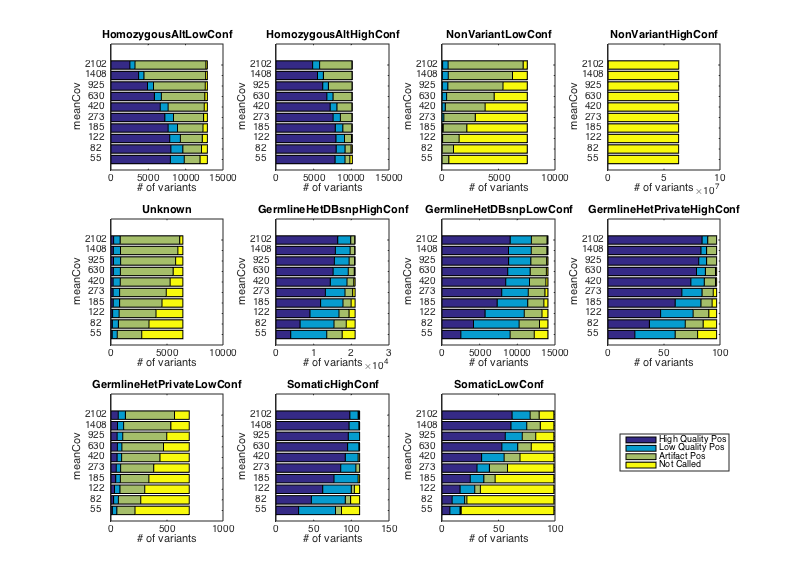

Supplement: Supplementary file 7 — Variant Quality Filtering By Sample. Shows the number of variants of each type, in each quality filtering category. Each graph represents a variant type, each bar represents a sample, and the color of the bar represents the number of variants in each quality category. High quality positions have a PT > 0.99. Low quality positions have a PT < 0.99 but PV > 0.99. Artifacts have a PV < 0.99 and non variants are not considered by the tumor only caller (NaN). (PNG 49 kb) [file 12920_2017_296_MOESM7_ESM.png]

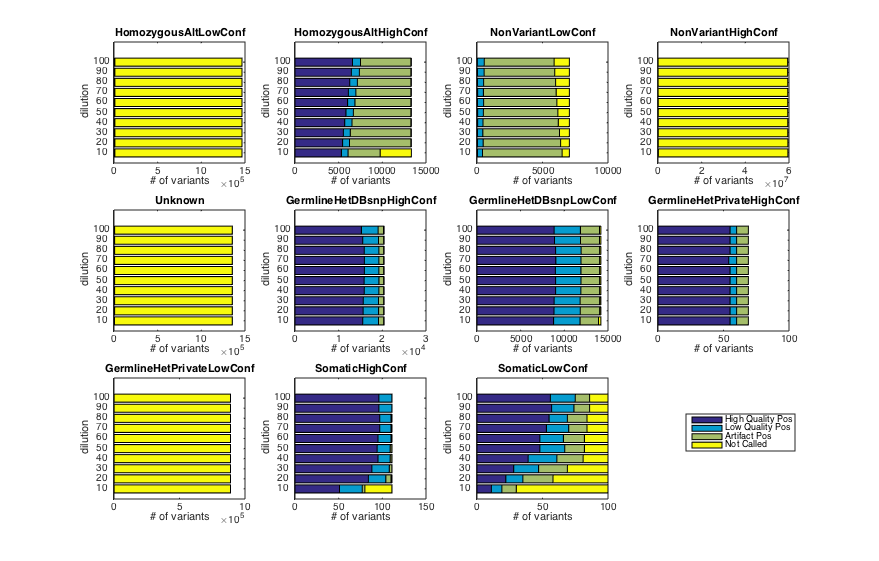

Supplement: Supplementary file 8 — Variant Quality Filtering Across Dilutions. Shows the number of variants of each type, in each quality filtering category. Each graph represents a variant type, each bar represents a dilution, and the color of the bar represents the number of variants in each quality category. High quality positions have a PT > 0.99. Low quality positions have a PT < 0.99 but PV > 0.99. Artifacts have a PV < 0.99 and non variants are not considered by the tumor only caller (NaN). (PNG 43 kb) [file 12920_2017_296_MOESM8_ESM.png]

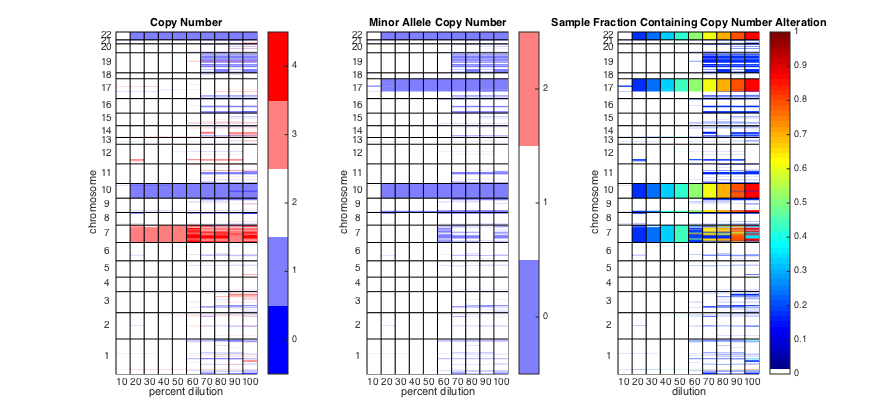

Supplement: Supplementary file 9 — Copy Number and Sample Fraction Across Dilutions. The copy number (left), minor allele copy number (center) and sample fraction of the copy number events (right) are plotted as heatmaps. (PNG 35 kb) [file 12920_2017_296_MOESM9_ESM.png]

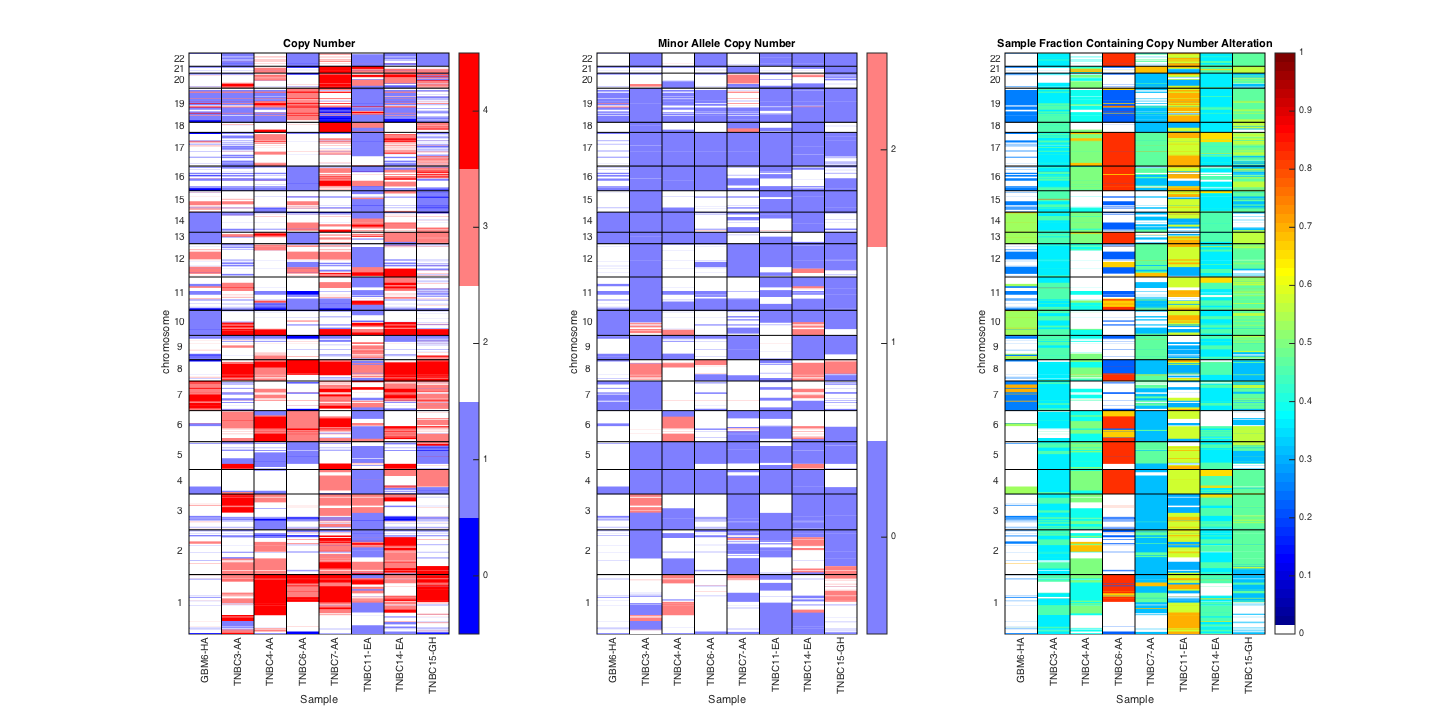

Supplement: Supplementary file 10 — Copy Number of and Sample Fractions Across Sample Set. The copy number (left), minor allele copy number (center) and sample fraction of the copy number events (right) are plotted as heatmaps. (PNG 68 kb) [file 12920_2017_296_MOESM10_ESM.png]

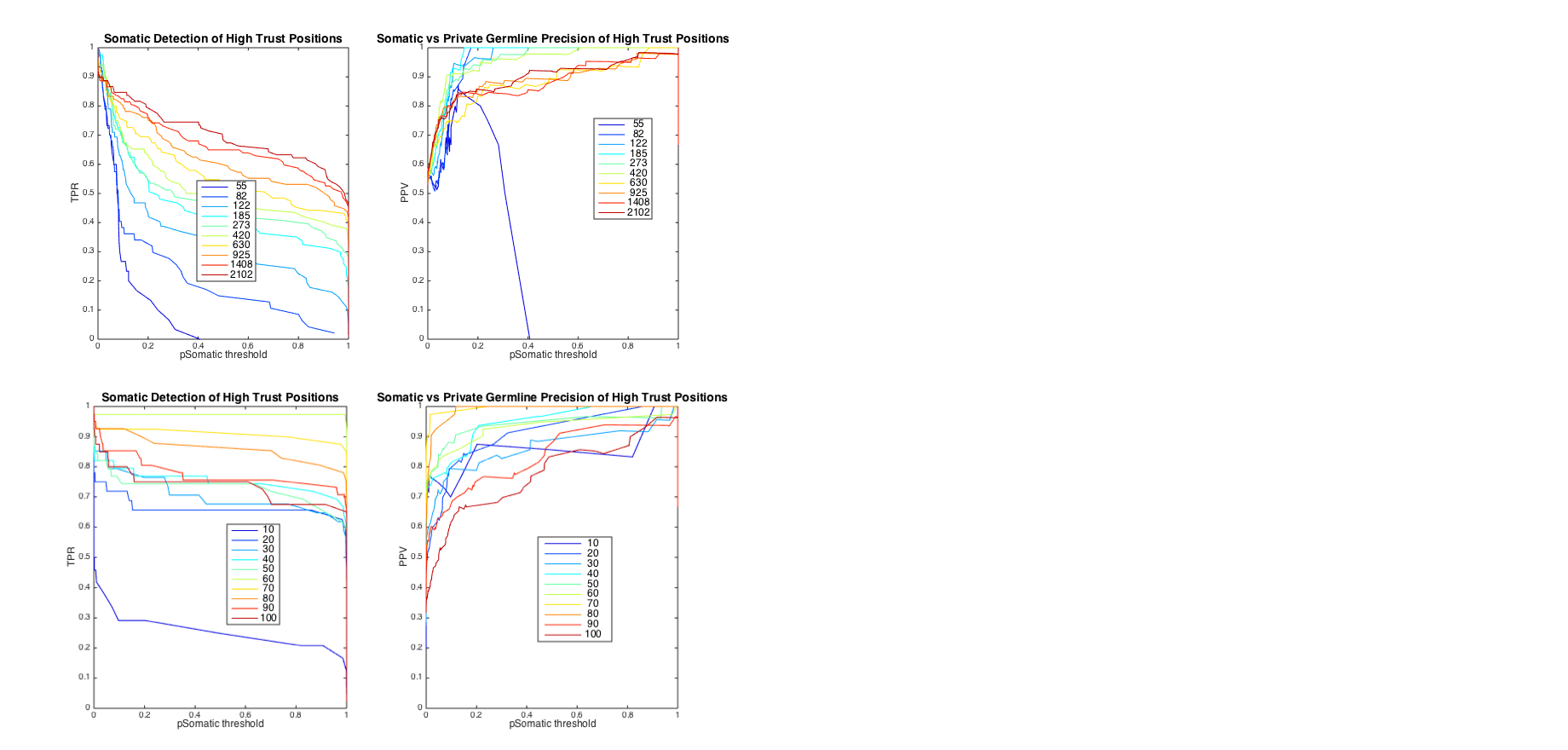

Supplement: Supplementary file 11 — Effect of Threshold on Sensitivity and Precision. The true positive rate (left) or positive predictive value (right) is plotted against the pSomatic threshold. Each line represents different mean target coverage (top) or dilution (bottom). Only high trust true somatic or private germline variants are included in this graph. As we would expect, the sensitivity decreases with the threshold, but the positive predictive value increases. We also find that higher coverage results in better sensitivity, but lower positive predictive value. At higher coverage, the threshold may be increased to improve the positive predictive value with less loss sensitivity. (PNG 185 kb) [file 12920_2017_296_MOESM11_ESM.png]
