# Supplementary material for: A method to reduce ancestry related germline false positives in tumor only somatic variant calling
Source: BMC Med Genomics. 2017 Oct 19;10:61. doi: 10.1186/s12920-017-0296-8 (PMC5649057; doi:10.1186/s12920-017-0296-8)
Supplement: Supplementary file 2 — Definition of True Variants. Describes the criteria for counting a variant as a true variant. Germline variants were called by haplotype caller, samtools, and freebayes. Somatic variants were called by Mutect, Seurat, and Strelka. (DOCX 15 kb) [file 12920_2017_296_MOESM2_ESM.docx]

# Table S1 Definition of True Variants

|  | Variant Type | Germline Callers | Somatic vs Matched Normals | Somatic vs. Reference | Has RS |
| --- | --- | --- | --- | --- | --- |
| HigHih Confidence | Germline Homozygous Alt | 1/1 with QUAL>30 in 3 of 3 | - | - | - |
|  | Germline Het DB | 0/1 with QUAL>30 in 3 of 3 | - | - | Yes |
|  | Germline Het Private | 0/1 with QUAL >30 in 3 of 3 | - | - | No |
|  | Somatic | 0/0 with QUAL>30 or  no call in 3 of 3 | SNVs: 3 of 3 callers  indels: 2 of 2 callers | SNVs: 3 of 3 callers  indels: 2 of 2 callers | - |
|  | Non Variant | 0/0 with QUAL>30 or  no call in 3 of 3 | 0 | 0 | - |
| Low Confidence | Germline Homozygous Alt | 1/1 with QUAL>30 in 1 or 2 | - | - | - |
|  | Germline Het DB | 0/1 with QUAL>30 in 1 or 2 | - | - | Yes |
|  | Germline Het Private | 0/1 with QUAL >30 in 1 or 2 | - | - | No |
|  | Somatic | 0/0 with QUAL>30 or  no call in at least 1 | At least 1 caller | At least 1 caller | - |
|  | Non Variant | 0/0 with QUAL>30 or  no call in 1 or 2 | 0 | 0 | - |
| Unknown | | Different genotypes with QUAL>30 | - | - | - |
|  |  | QUAL <30 in 3 of 3 | - | - | - |
|  |  | 0/1 with QUAL>30 in at least 1 caller | At least 1 caller | At least 1 caller | - |
