# Supplementary material for: A method to reduce ancestry related germline false positives in tumor only somatic variant calling
Source: BMC Med Genomics. 2017 Oct 19;10:61. doi: 10.1186/s12920-017-0296-8 (PMC5649057; doi:10.1186/s12920-017-0296-8)
Supplement: Supplementary file 4 — Filtering Metrics. The criteria used to initially classify a variant in the training set for the quadratic discriminant model. (DOCX 14 kb) [file 12920_2017_296_MOESM4_ESM.docx]

# Table S2 Filtering Metrics

| Metric | Criteria for PASS | Criteria for Reject |
| --- | --- | --- |
| Percentage of Bases with MQ>MQ_min_ and BQ>BQ_min_ | >80% | <70% |
| Percentage of Bases that support the A or B allele | >95% | <90% |
| Minimum percentage of reads from forward or reverse strand | >1% | <0.1% |
| Minimum average mapping quality of reads supporting A or B allele | >35 | <30 |
| Minimum average base quality of bases supporting A or B allele | >25 | <20 |
| Maximum average percentage of mismatches in reads supporting A or B alleles | <2.5% | >5% |
| Minimum average distance from either end of sequence of A or B allele | >30 | <10 |
| Difference in average percentage of forward strand between A and B allele | <10% | >20% |
| Difference in average base quality between A and B alleles | <5 | >10 |
| Difference in average mapping quality between A and B alleles | <10 | >15 |
| Difference in average percentage of mismatches between A and B alleles | <1% | >2% |
| Difference in average read position between A and B alleles | <10 | >20 |
| Quality score of position from unmatched controls | >30 | <10 |
| Mean quality score in region | >20 | <10 |
