# Supplementary material for: A method to reduce ancestry related germline false positives in tumor only somatic variant calling
Source: BMC Med Genomics. 2017 Oct 19;10:61. doi: 10.1186/s12920-017-0296-8 (PMC5649057; doi:10.1186/s12920-017-0296-8)
Supplement: Supplementary file 5 — One thousand Genomes Population Codes. Abbreviations used to describe the populations from the 1000 Genomes Project. (DOCX 17 kb) [file 12920_2017_296_MOESM5_ESM.docx]

## Table S3a 1000 Genomes Population Codes.

| Population Code | Population Description | Super Population Code |
| --- | --- | --- |
| CHB | Han Chinese in Bejing, China | EAS |
| JPT | Japanese in Tokyo, Japan | EAS |
| CHS | Southern Han Chinese | EAS |
| CDX | Chinese Dai in Xishuangbanna, China | EAS |
| KHV | Kinh in Ho Chi Minh City, Vietnam | EAS |
| CEU | Utah Residents (CEPH) with Northern and Western European Ancestry | EUR |
| TSI | Toscani in Italia | EUR |
| FIN | Finnish in Finland | EUR |
| GBR | British in England and Scotland | EUR |
| IBS | Iberian Population in Spain | EUR |
| YRI | Yoruba in Ibadan, Nigeria | AFR |
| LWK | Luhya in Webuye, Kenya | AFR |
| GWD | Gambian in Western Divisions in the Gambia | AFR |
| MSL | Mende in Sierra Leone | AFR |
| ESN | Esan in Nigeria | AFR |
| ASW | Americans of African Ancestry in SW USA | AFR |
| ACB | African Caribbeans in Barbados | AFR |
| MXL | Mexican Ancestry from Los Angeles USA | AMR |
| PUR | Puerto Ricans from Puerto Rico | AMR |
| CLM | Colombians from Medellin, Colombia | AMR |
| PEL | Peruvians from Lima, Peru | AMR |
| GIH | Gujarati Indian from Houston, Texas | SAS |
| PJL | Punjabi from Lahore, Pakistan | SAS |
| BEB | Bengali from Bangladesh | SAS |
| STU | Sri Lankan Tamil from the UK | SAS |
| ITU | Indian Telugu from the UK | SAS |

## Table S3b 1000 Genomes Super Population Codes.

| Code | Super Population |
| --- | --- |
| AFR | African |
| AMR | Ad Mixed American |
| EAS | East Asian |
| EUR | European |
| SAS | South Asian |
